# On–Off Childhood? A Rapid Review of the Impact of Technology on Children’s Health

**DOI:** 10.3390/healthcare13141769

**Published:** 2025-07-21

**Authors:** Diana Borges, Inês Pinto, Octávio Santos, Ivone Moura, Iara Rafaela Ferreira, Ana Paula Macedo, Adriana Taveira

**Affiliations:** 1 School of Health, University of Trás-os-Montes and Alto Douro (UTAD), 5000-801 Vila Real, Portugal; dianapatriciasilvaborges@gmail.com (D.B.); inespinto541@gmail.com (I.P.); octavio.vigariosantos@gmail.com (O.S.); 2 Local Health Unit of Trás-os-Montes and Alto Douro (ULSTMAD), Paediatrics Outpatient Clinic, Chaves Hospital, 5400-279 Chaves, Portugal; ivonebrancomoura@gmail.com; 3 Health Sciences Research Unit: Nursing (UICISA: E), Nursing School of Coimbra, 3046-851 Coimbra, Portugal; b14265@ese.uminho.pt (I.R.F.); amacedo@ese.uminho.pt (A.P.M.)

**Keywords:** paediatric nursing, digital technology, digital health, child development, screen time

## Abstract

**Background/Objectives**: The use of digital technologies among children and adolescents has been increasing exponentially, raising concerns about the potential impacts on physical, mental, cognitive, educational, and social development. Understanding these effects is key to informing clinical and educational practices and public policies that promote digital wellbeing in childhood and adolescence. The main objective of this study was to map the latest available scientific evidence on the patterns of digital technology use by children and adolescents and its main impact, identifying risk factors, opportunities, and strategies for promoting digital wellbeing. **Methods**: A rapid review was carried out following the Joanna Briggs Institute (JBI) guidelines. Quantitative, qualitative, and mixed studies published between 2020 and 2025, in Portuguese or English, that addressed the use of digital technologies by children and adolescents were included. The assessment of methodological quality was based on JBI’s Critical Appraisal Tools. **Results**: Ten studies from diverse contexts showed an association between excessive screen time and risks of sedentary lifestyles, sleep disorders, anxiety, depression, attention difficulties, and low academic performance. Occasional benefits arose with adult mediation and educational use; parental mediation and socioeconomic background were key factors. **Conclusions**: The use of digital technologies is a complex and multifactorial phenomenon that requires integrated approaches; the promotion of digital literacy, public policies for equitable access to quality digital resources, and longitudinal and intercultural studies are recommended to clarify causal relationships and adapt interventions to local contexts.

## 1. Introduction

The concept of the “Screen Generation” refers to today’s cohort of children and adolescents who are growing up surrounded by digital technologies, with early, intense, and prolonged exposure to devices such as smartphones, tablets, and computers [1,2]. This constant connectivity is reshaping how young people communicate, learn, and spend their leisure time, while also influencing key aspects of their physical, cognitive, and socio-emotional development.

When it comes to the “excessive use” of digital technology, there is no universally agreed-upon threshold. However, leading health authorities such as the World Health Organisation (WHO) and the American Academy of Paediatrics offer operational definitions. For instance, ref. [3] recommends that children aged from 2 to 5 years should have no more than one hour of sedentary screen time per day, and children under 2 should avoid screens altogether. For older children and adolescents, while there are no strict time limits, “excessive use” is generally defined as screen time that disrupts sleep, physical activity, social interactions, or academic performance [3]. In this study, we use the term “excessive use” to refer to digital engagement that exceeds these recommended limits or negatively impacts essential aspects of health and daily life.

The prevalence of mobile technology use among children and adolescents has grown steadily, with internet access beginning at increasingly younger ages [4]. While digital technologies offer valuable opportunities for digital literacy, social inclusion, and educational innovation, the risks associated with their overuse are a growing concern for researchers, educators, and health professionals alike.

A substantial body of scientific literature highlights strong associations between excessive screen time and a range of adverse health outcomes in children. These include disruptions to sleep, increased sedentary behaviour, visual problems, and symptoms of anxiety and depression, as well as negative effects on social and academic skills [5,6,7]. Notably, one of the most discussed consequences is the heightened risk of attention deficit and hyperactivity disorder (ADHD) among children and adolescents. Recent studies, such as [1], have shown that prolonged and unregulated exposure to digital technologies is linked to increased inattention, impulsivity, and challenges with self-control—core features of ADHD. Their findings suggest that even one hour of daily screen use can be associated with lower curiosity, reduced emotional stability, and greater difficulty completing tasks. Adolescents with higher screen time are also more likely to be diagnosed with psychological and behavioural disorders, including anxiety, depression, and attention deficits [1]. While these relationships are complex and multifaceted, evidence suggests that excessive screen use can exacerbate pre-existing vulnerabilities or contribute to the development of attention problems, especially when combined with poor sleep and limited opportunities for physical and social activity. Importantly, these effects can extend beyond the immediate term, potentially influencing children’s and adolescents’ overall growth and development over time.

Recent systematic reviews and meta-analyses have further quantified these health effects. For example, ref. [5] found that higher screen time is associated with an increased risk of obesity, depressive symptoms, and poorer educational outcomes. These impacts are not uniform: younger children are more prone to developmental delays, while adolescents are at a greater risk for sleep disturbances and mental health issues. Gender differences have also emerged, with girls more likely to experience anxiety and depressive symptoms linked to social media use and boys more affected by gaming-related problems. Socioeconomic status adds another layer, as children from lower-income families may have less access to digital literacy resources and parental guidance [5,8].

Within clinical settings, there is a growing recognition of the link between problematic technology use in paediatric populations and various complications in physical, cognitive, and emotional development. This underscores the urgent need to better understand and address this phenomenon, not only to manage emerging harms, but also to promote healthier, more balanced ways of interacting with technology.

Despite a wealth of research on this topic, important gaps remain. Many studies are methodologically diverse, culturally specific, and do not always explore the complexity of moderating factors such as quality of use, content consumed, and family context [9]. Furthermore, the rapid pace of technological change demands continual updates to the evidence base, lest it quickly become outdated. In this context, rapid reviews have emerged as essential tools for efficiently synthesising the best available scientific evidence, supporting informed decisions in education and public health [10].

Given these considerations, this study aims to conduct a rapid review of the literature, guided by the following central question: “What is the impact of technology use on the health of children and adolescents?” The primary objective is to map the latest scientific evidence regarding the patterns of digital technology use among children and adolescents and to identify the main health impacts, risk factors, opportunities, and strategies for promoting digital wellbeing.

The concept of digital wellbeing, while increasingly discussed, is multifaceted and warrants deeper reflection. Digital wellbeing encompasses emotional, cognitive, social, and ethical dimensions [11]. Emotionally, it involves managing online stress and maintaining positive affect; cognitively, it relates to attention regulation and critical thinking in digital environments; socially, it concerns the quality of both online and offline relationships; and ethically, it refers to responsible and safe technology use [12,13]. For children and adolescents, digital wellbeing is especially complex, as their developmental stage makes them more vulnerable to online risks and less equipped for self-regulation [13]. Current debates highlight challenges such as cyberbullying, exposure to inappropriate content, and the tension between digital opportunities and risks [12,13]. Promoting digital wellbeing, therefore, requires not only limiting screen time, but also fostering digital literacy, resilience, and ethical engagement with technology [11].

To achieve these aims, the following specific objectives are outlined: (i) to identify the effects of excessive digital technology use on the physical, cognitive, and emotional development of children and adolescents; (ii) to systematise the implications of technology use for children’s and adolescents’ education; and (iii) to identify intervention strategies for parents, health professionals, educators, and teachers.

Ultimately, this approach seeks to mitigate the negative impacts of digital technology on children and young people by synthesising the current state of knowledge. This mapping will serve as a foundation for developing innovative practices that promote sustained health and digital literacy, tailored to the contemporary challenges faced by the digital youth generation.

## 2. Materials and Methods

This study consists of a rapid literature review, a form of evidence synthesis which, although based on the methodological principles of systematic reviews, includes adaptations aimed at maximising the time and resources available. Recognised for its ability to offer agile and well-founded answers to emerging questions, this approach has been gaining prominence, especially in the areas of health and education, for its practical usefulness and commitment to scientific quality standards [10].

The review was conducted based on the methodological guidelines of the WHO, the Joanna Briggs Institute (JBI), and the Cochrane Rapid Reviews Methods Group and was adjusted to the reality and specific resources of the current project. The PICO (Population, Intervention, Comparison, Outcome) model, widely applied to health research, was used to formulate the research question, define the eligibility criteria, and build the research strategy. This model was also explicitly integrated into the narrative structure of the review, guiding each stage of the methodology, from data extraction to synthesis.

The aim was to strike a balance between the methodological simplification inherent in the rapid review format, particularly in the number of databases consulted, the period of analysis, and the duplication of processes, and the rigour that is essential to guarantee the validity and applicability of the results obtained.

### 2.1. Eligibility Criteria

To guarantee the relevance and timeliness of the data included in the review, strict inclusion criteria were defined, as follows:

- Empirical studies (quantitative, qualitative, and mixed methods);
- Studies with populations between the ages of 2 and 18;
- Articles published between 2020 and 2025;
- Publications in Portuguese or English;
- Studies with full text available;
- Research addressing the impacts on physical, mental, and emotional health and/or educational aspects associated with the use of digital technologies with internet access.

The initial eligibility criteria specified an age range of “0–18”, but the inclusion criteria were subsequently refined to encompass participants from the age of one and above. This adjustment is indicative of the recognition that meaningful engagement with digital technologies typically commences after infancy. Infants under 24 months of age may be exposed to screens passively, for example, via family media use. However, studies focusing exclusively on this age group were not eligible for inclusion unless they also included broader child populations. Concerning the upper limit, the age of “18 years” was maintained to align with the definition of adolescence provided by the WHO. This was also chosen to encompass a wider spectrum of studies that address youth digital engagement. While numerous studies have centred on children under the age of 15, including older adolescents, they have facilitated the identification of transitional behaviours and late developmental impacts pertinent to digital wellbeing.

The delimitation of the time interval between 2020 and 2025 is justified by the need to gather recent evidence that is sensitive to the contemporary technological and social context. Since 2020, with the onset of the COVID-19 pandemic, there has been a global intensification of dependence on mobile devices and digital platforms, especially among children and adolescents, both in educational and recreational contexts [14]. This generational milestone has translated into substantial changes in digital habits and relationships with technology, which is widely reflected in the most recent scientific literature. Thus, the inclusion of studies published in this period guarantees the timeliness, relevance, and practical applicability of the data summarised in this review.

The exclusion criteria were as follows:

- Duplicate articles;
- Systematic, integrative, narrative, or other types of secondary synthesis reviews;
- Publications without explicit methodology or results;
- Grey literature (theses, dissertations, and non-peer-reviewed reports), which was excluded to ensure methodological rigour and the inclusion of peer-reviewed, high-quality sources. To determine peer-review status, the indexing of the journal (e.g., inclusion in PubMed, Web of Science, or Scopus), journal website documentation, and publisher information were verified.

### 2.2. Research Strategy

The literature search was conducted in a structured way to identify relevant studies on the impact of the use of digital technologies and devices with internet access on the health and education of children and adolescents. The search strategy was based on the PICO model (see Table 1), and the keywords were adapted to the terminology and syntax of each database.

The selection of databases was guided by their thematic relevance, disciplinary coverage, and accessibility. PubMed was selected for its robust indexing of health sciences literature; SciELO and B-On were included due to their emphasis on Ibero-American and open-access scientific production. The full search strategies for each database (including Boolean combinations and filters applied) are provided as Supplementary Material to enhance transparency and reproducibility.

The following is an example of the strategy applied in PubMed: (“children” OR “adolescents”) AND (“technology” OR “mobile devices” OR “smartphone” OR “internet”) AND (“effects”) OR (“Consequences”) OR (“Impact”) AND (“health” OR “Development” OR “education” OR “physical well-being” OR “emotional well-being”) AND (“Recommendations” OR “Strategies” OR “Good Practices”).

### 2.3. Study Selection Process

Studies were selected in a systematic and structured way, following the methodological principles recommended for rapid reviews, with appropriate adaptations to the context and available resources [3,10]. The process was carried out in three sequential stages, ensuring the traceability and reproducibility of the procedures adopted.

In the first stage, the results obtained from the databases were initially screened by reading the titles and abstracts. At this stage, the previously defined inclusion and exclusion criteria were applied to eliminate irrelevant studies, duplicates, or those that did not fulfil the minimum formal requirements.

The second stage consisted of a full reading of the pre-selected articles to confirm their eligibility based on methodological suitability, clarity of objectives, and thematic relevance to the research question. This reading also made it possible to assess the depth of the data presented, the framing of the results, and their suitability for the scope of the review.

In the third stage, the final assessment of the studies to be included in the synthesis was carried out. This analysis also considered the methodological quality of the studies based on the application of the JBI checklist, as described in the following section. The studies that met the minimum quality criteria were included in the narrative extraction and synthesis matrix.

The entire selection process was conducted independently by two reviewers. A record was kept of all decisions, and any disagreements were resolved through consensus after discussion. Although no software was used to manage the screening process, a manual spreadsheet was employed to track inclusion decisions and ensure consistency.

**Figure 1:**
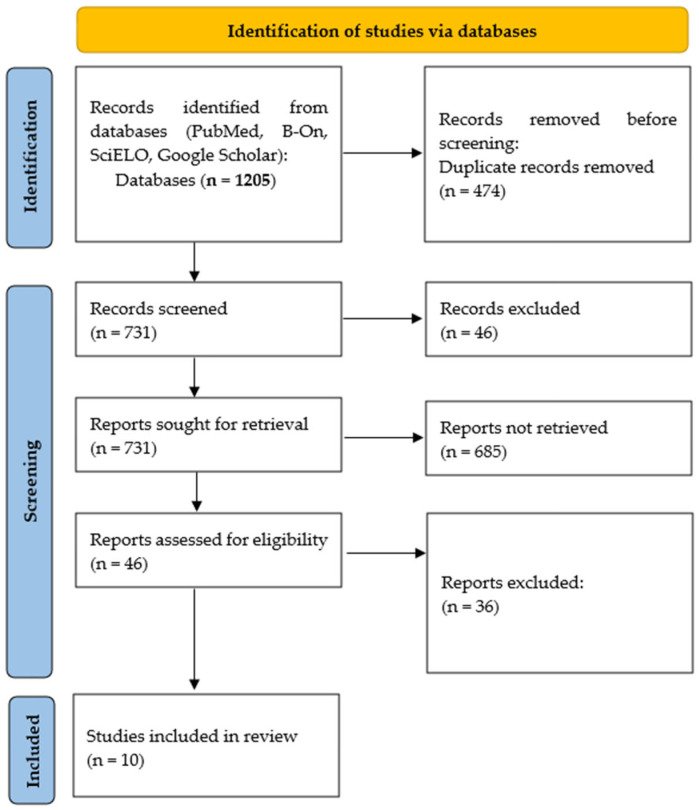
PRISMA flowchart (adapted to rapid reviews).

The flowchart of the selection process was drawn up according to the guidelines of the PRISMA (Preferred Reporting Items for Systematic Reviews and Meta-Analyses) model adapted to rapid reviews, illustrating the number of records identified, screened, read in full, and finally included in the synthesis (see Figure 1). In addition to the flowchart, the PRISMA checklist for rapid reviews was used as a reporting guideline to structure the methodology, selection process, and presentation of results, ensuring transparency and alignment with international standards. This diagram provides a clear and transparent visualisation of the steps taken throughout the review process.

### 2.4. Evaluation of Methodological Quality

The critical assessment of the methodological quality of the studies included in this review was conducted using tools developed by the JBI, internationally recognised as one of the main references in evidence synthesis and methodological assessment in health [15]. The use of validated appraisal tools is a fundamental step in integrative and rapid reviews, ensuring transparency, internal validity, and confidence in the synthesised results.

The JBI Critical Appraisal Tools appropriate to the methodological design of each study included were used, as follows:

- The checklist for quantitative descriptive studies (JBI Critical Appraisal Checklist for Analytical Cross-Sectional Studies) (see Table 2);
- The checklist for qualitative studies (JBI Critical Appraisal Checklist for Qualitative Research) (see Table 3);
- The JBI Critical Appraisal Checklist for Mixed-Methods studies (see Table 4).

Each checklist is made up of a set of criteria that assess, among other things, the following:

- The clarity of the research question;
- The adequacy of the inclusion criteria;
- The validation of data collection instruments;
- The treatment of confounding factors;
- The relevance of statistical analyses or qualitative interpretation.

The studies were independently assessed by two reviewers. Each item was assigned one of the following categories: “Yes”, “No”, “Unclear”, or “Not applicable”, by the JBI guidelines. Disagreements between reviewers were resolved by consensus after discussion of the criteria. The final score for each study resulted from the sum of the positively assessed items, allowing an overall qualitative classification to be given, as follows:

- High quality (≥75% of criteria met);
- Moderate quality (50–74%);
- Low quality (<50%).

The application of the JBI’s Critical Appraisal Tools to the quantitative methodology studies included in this review showed a consistently high methodological quality. Of the four quantitative studies assessed, all obtained the maximum scores in their respective assessment tools, meeting 100% of the established criteria.

Three studies [16,17,18] adopted a quantitative cross-sectional design and were assessed using the JBI Critical Appraisal Checklist for Analytical Cross-Sectional Studies, scoring 10/10 items. These studies demonstrated methodological rigour in the definition of the research question, adequacy of the inclusion and exclusion criteria, validation of the data collection instruments, control of confounding factors, and relevance of the statistical analyses carried out.

The study by [19], characterised by a cohort design, was assessed using the JBI Critical Appraisal Checklist for Cohort Studies, achieving the maximum score of 11/11 criteria. This study showed particular methodological robustness in the longitudinal follow-up of participants, the clear definition of exposure and outcomes, and the control of confounding variables over time.

It should be noted that the study by [17] stood out due to the considerable size of the sample (n = 2440), which gives a greater statistical power to the analyses and robustness to the results presented. All the quantitative studies were classified as “high quality”, meeting more than 75% of the methodological criteria established by the JBI, which reinforces confidence in the results and conclusions derived from this category of evidence.

The unanimity in the high-quality classification of the quantitative studies contributes significantly to the internal validity of this review, providing a solid basis for the synthesis and interpretation of the results related to the impacts of the use of digital technologies by children and adolescents.

The evaluation of the qualitative methodology studies, as shown in Table 3, was carried out using the JBI Critical Appraisal Checklist for Qualitative Research, allowing the methodological rigour and transparency of the qualitative research processes included in this review to be gauged.

Of the four studies assessed, three were classified as high quality, having met more than 75% of the criteria established by the JBI instrument. The study by [21] obtained the highest score, with 10 of the 11 criteria met (90.9%), demonstrating a high rigour in the definition of the research question, description of the context, and adequacy of the data collection and analysis methods, as well as in explaining the interpretations and conclusions. The studies by [22,23] also showed a robust methodological quality, meeting 81.8% of the criteria (9/11), standing out for their clarity in presenting the methodological procedures and consistency in analysing and interpreting the qualitative data.

On the other hand, the study by [20] was classified as being of moderate quality, having met 72.7% of the criteria (8/11). This result reflects some methodological limitations, namely in the description of the data validation procedures and in explaining the position of the researchers, aspects that may impact transparency and confidence in the conclusions presented.

Overall, the evaluation shows that most of the qualitative studies included are of high methodological quality, reinforcing the robustness and credibility of the qualitative evidence synthesised in this review. The systematic use of the JBI appraisal instruments made it possible to identify strengths and limitations, contributing to a critical and reasoned appraisal of the qualitative results.

The evaluation of the mixed-methodology studies, presented in Table 4, shows a high level of methodological rigour among the studies included. Both studies analysed [24,25] were assessed using the JBI Critical Appraisal Checklist for Mixed-Methods Studies and obtained the maximum score (9 out of 9 criteria, corresponding to 100%). This assessment translates into an overall rating of high quality, as both studies met more than 75% of the criteria defined by the instrument.

The study by [24] stands out for presenting an in-depth discussion of parental mediation strategies, making a relevant contribution to understanding the mechanisms for protecting and managing digital use in a family context. The study by [25] has a large quantitative sample (n = 582) and provides clear correlations between variables, complemented by robust qualitative evidence, which reinforces the validity of the results presented.

To summarise, both mixed-methodology studies included in this review demonstrate a high methodological quality, making a significant contribution to the robustness of the evidence on the impact of digital technologies and parental mediation strategies in different contexts.

### 2.5. Data Synthesis and Analysis

The data was extracted using a table previously structured by the research team, allowing the main information from each included study to be systematised. The table included the following components, among others: author, year of publication, country of origin, type of study, sample characteristics, study objective, and main conclusions. This approach ensured the homogeneity of the data collection process and facilitated the matching of studies.

The data was analysed using a narrative synthesis strategy, appropriate for rapid reviews and studies with different methodological designs. This approach allows for the integration of qualitative and quantitative evidence, articulating the findings along common interpretative lines.

The analysis process was based on a comprehensive reading and analysis of the results of the studies included, identifying incidences, contrasts, trends, and individual contributions, which were then organised into thematic categories. The definition of these categories resulted from an inductive thematic analysis, respecting the empirical content of the studies and their relevance to the research question. The four main categories were as follows:

1. Patterns of digital technology use, including data on the frequency and duration of mobile device use, types of devices most used (e.g., smartphones and tablets), preferred platforms (e.g., social networks, games, and educational platforms), and associated behaviours (night-time use and digital multitasking). Variations according to age, gender, socioeconomic background, and parental supervision were also taken into account.
2. Impacts on physical and emotional health—aggregating the results relating to the effects of excessive technology use on physical health (e.g., sleep disturbances, sedentary lifestyles, muscle pain, and eyestrain) and mental and emotional health (e.g., anxiety, irritability, depressive symptoms, isolation, and digital addiction). This category also includes findings on the perceptions of young people themselves and their carers regarding the impact of digital use on their wellbeing.
3. Impacts on cognitive and educational development—referring to reported effects on attention, memory, academic performance, problem-solving skills, self-regulation, and school motivation. This category also includes studies analysing the correlation between screen time and school performance, as well as the role of digital technologies in formal and informal learning contexts.
4. Mediation strategies and the promotion of digital wellbeing—compiling evidence on the strategies adopted by parents, educators, and health professionals to regulate the use of technology by children and adolescents. This category includes parental mediation practices (active, restrictive, and joint), educational interventions in digital literacy, clinical recommendations, and institutional policies aimed at promoting healthy digital behaviours.

Each thematic category was developed based on the contributions of the studies included, maintaining rigour in the interpretation of the data and respecting the methodological limits identified. The diversity of the geographical and methodological contexts of the analysed studies allowed for a cross-sectional reading of the phenomenon, favouring the identification of regularities and contextual specificities that enrich the understanding of the impact of technology on children’s health and education.

## 3. Results

A total of 10 studies published between 2020 and 2025 were included in the review, covering different geographical, methodological, and population contexts (see Table 5). Analysing the included studies reveals a diverse geographical distribution: 20% of the studies were carried out in Brazil, 20% in the United States of America, 20% in Indonesia, 20% in Croatia, 10% in Chile, 10% in Nigeria, and 10% in Turkey. In methodological terms, 60% of the studies adopted a cross-sectional quantitative approach, while 40% used qualitative or mixed methods.

Regarding the target population, 50% of the studies focused on children and adolescents, 30% involved both parents and children, and 20% included health or education professionals as secondary participants.

The thematic categories presented in this review were developed through an inductive content analysis. Following the extraction of the primary findings from each study using a structured data matrix, the researchers conducted a comprehensive review to identify recurring topics, contrasts, and patterns across the studies. These elements were subsequently organised into broader thematic categories. Despite the absence of software applications such as NVivo or ATLAS.ti in the data extraction or synthesis process, the procedure was meticulously guided by a manual thematic coding framework that was applied in a consistent manner across all studies by two independent reviewers.

To enhance the traceability between the categories and the supporting evidence, Table 6 has been incorporated. The following table offers a concise overview of the primary thematic categories, with each category accompanied by the relevant studies that contributed to its identification and the key findings that emerged.

The main emerging thematic categories were as follows:

- In total, 10% of the studies highlighted that 94.5% of children use digital devices, with 63% having more screen time than recommended.
- In total, 20% of the studies identified the smartphone as the preferred device for games and videos, with entertainment as the main motivation.
- In total, 10% of the studies showed that the night-time use of technology compromises sleep hygiene and school performance, with sleep deprivation as a central mediating factor.
- In total, 10% of the studies reported that increased screen time is associated with inappropriate eating behaviours, socialisation difficulties, and a negative impact on language development.
- In total, 10% of the studies found a significant association between screen time and mental health symptoms such as depression and behavioural changes in adolescents.
- In total, 10% of the studies found that restrictive parenting strategies were associated with lower digital risk, while active mediation was related to higher online use.
- In total, 20% of the studies addressed the use of digital devices as a parental strategy to promote calm and occupation, recognising risks such as addiction and emotional and cognitive impacts.

A comparative analysis between studies carried out in emerging countries (Brazil, Chile, Indonesia, Nigeria, and Turkey) and developed countries (the United States and Croatia) revealed significant differences in terms of thematic focus, methodology, and participant profile. In emerging countries, the majority of studies (approximately 70%) focused on the physical and behavioural risks associated with technology use, while in developed countries, the focus was predominantly on participants’ mental health (100%). In methodological terms, there was a greater prevalence of qualitative approaches in emerging countries (around 57%), while in developed countries, quantitative methodologies were predominant (67%). Regarding the profile of the participants, the studies from emerging countries mostly involved parents and children together (71%), in contrast to the studies from developed countries, in which all the participants were adolescents assessed individually. These results suggest that socioeconomic and cultural context influences not only the objectives of the research, but also the methodological strategies employed and the composition of the samples, reflecting different priorities and local challenges in the study of technology use by children and adolescents.

The data reveals transnational patterns, such as the parental use of devices as an emotional regulation strategy, identified in 80% of the studies. However, there are regional differences: while South American studies emphasise socioeconomic determinants, European and North American studies highlight clinical correlations. This methodological and contextual heterogeneity limits generalisation but enriches the multifactorial understanding of the phenomenon.

In summary, there is a predominance of quantitative studies and a strong presence of qualitative research, reflecting the complexity of the phenomenon and the need for multifactorial approaches. The recurring thematic categories include patterns of technological use, parental mediation, and impacts on child development and mental health, with cross-cutting trends and regional specificities being identified that are relevant to understanding the use of technology in childhood and adolescence.

The narrative analysis allowed the results to be systematised into the following six main thematic categories, previously defined in the data analysis phase: (a) patterns of digital technology use; (b) impacts on physical health; (c) impacts on mental and emotional health; (d) impacts on cognitive development; (e) impacts on educational and social development; and (f) factors that mediate and promote digital wellbeing. These categories reflect the dimensions most frequently addressed in the included studies and allow for an integrated, cross-sectional reading of the effects of the use of technologies with internet access by children and adolescents. Table 6 summarises the main evidence from the included studies, organised by predominant thematic category.

Although the analysis of the studies included shows a great diversity of contexts and methodologies, it reveals consistent patterns in all thematic categories. Regarding patterns of digital technology use, it can be seen that the use of screens starts early, with children accessing the internet between the ages of 1 and 5, and in some studies, more than 80% already have a device [21]. The average daily exposure time ranges from 4 to 8 h, with smartphones, television, online games, and social networks predominating [16,20]. The most frequently mentioned motivations include entertainment, communication, learning, and, in adolescents, the need for social connection and belonging [17,18].

Concerning impacts on physical health, most studies associate excessive screen time with a sedentary lifestyle, obesity, sleep disorders, headaches, visual fatigue, and, to a lesser extent, changes in diet and increased blood pressure [16,20,21]. Parents report concern about the reduction in time dedicated to physical activity and sleep, factors that remain associated with physical symptoms even after adjusting for other variables [20].

Regarding impacts on mental and emotional health, the association between prolonged use of technology and symptoms of anxiety, irritability, frustration, mood swings, and, in some cases, depressive symptoms and challenging behaviour stands out [18,20,21]. The risk of digital addiction, social isolation, and lower self-esteem is particularly emphasised in adolescents, especially when there are negative experiences online, such as cyberbullying or social comparison [17].

In the area of impacts on cognitive development, studies show that educational apps can benefit vocabulary and literacy, but excessive and unsupervised consumption of digital content tends to harm attention, language, creativity, and academic performance [16,20,21].

As for impacts on educational and social development, excessive use of technology is associated with a poorer school performance, less family interaction, and socialisation difficulties [17,18]. There is a growing preference for online communication over face-to-face communication, which can jeopardise the development of social skills, empathy, and emotional self-regulation [20]. Exposure to social risks, such as interaction with strangers and inappropriate content, is also a recurring concern [17,21].

Finally, with regard to the factors that mediate and promote digital wellbeing, the evidence reinforces the central roles of parental supervision, open communication, and family involvement in promoting the balanced and safe use of technology [16,20]. Active mediation strategies, the encouragement of alternative activities, and positive parental role models appear to be protective factors, while the imposition of rigid rules, without dialogue, can be associated with more dysfunctional use patterns [18,20]. In addition, cultural, technical, and sociodemographic factors modulate the observed impacts, suggesting the need for contextualised approaches [21].

**Figure 2:**
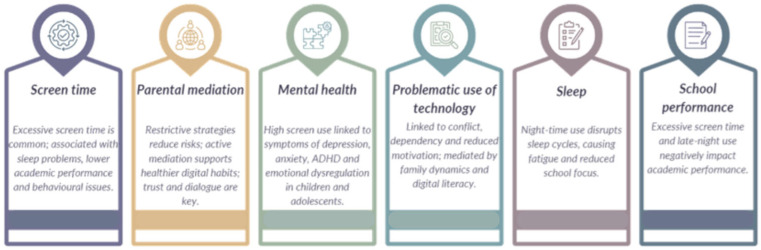
This figure summarises the main thematic categories identified in the review, including screen time, potential mediation, mental health, problematic technology use, sleep, and school performance. Adapted from [12,13,14,15,16,17,18,20,21].

Figure 2 provides a visual summary linking the main thematic categories to the corresponding studies and key findings, facilitating the traceability of the evidence.

## 4. Discussion

This rapid review shows that the use of digital technologies by children and adolescents is a multifaceted phenomenon, with impacts that span different dimensions of physical, cognitive, emotional, social. and educational development. The results summarised here dialogue with a growing body of international and national literature, reinforcing trends but also revealing important nuances for a critical understanding of the subject.

The predominance of quantitative studies of a high methodological quality (100% of the JBI criteria met) reinforces the robustness of the associations identified between excessive screen time and risks to physical health (sedentary lifestyle and sleep disorders) and mental health (anxiety and social isolation). These results are in line with recent systematic reviews, such as [1], which associated smartphone use with increases in depressive symptoms in adolescents, and the meta-analysis in [5], which confirmed significant correlations between screen time and childhood obesity.

The geographical heterogeneity of the studies (Brazil, USA, Indonesia, Nigeria, Croatia, and Turkey) made it possible to identify critical contextual differences. For example, while research in emerging countries highlighted socioeconomic determinants (e.g., unequal access to devices and poor parental mediation), studies in developed countries emphasised clinical correlations (e.g., ADHD and depression). This dichotomy reflects structural disparities, suggesting that universal interventions may be less effective than approaches adapted to local realities.

### 4.1. Regional, Cultural, and Socioeconomic Differences

Patterns of digital technology use among children and adolescents are deeply shaped by regional, cultural, and socioeconomic factors. Our review found that in high-income countries, concerns often focus on overuse, digital addiction, and exposure to harmful content, while in low- and middle-income regions, challenges include unequal access to devices, digital divides, and limited parental digital literacy [8,19]. These findings are consistent with large-scale studies showing that children from lower-income families and minority backgrounds tend to have higher screen time, often due to reduced access to safe outdoor spaces and fewer extracurricular opportunities, which can lead to increased reliance on digital entertainment [19]. Moreover, lower parental education is associated with a higher total screen time, and these patterns persist across different screen modalities. The intersection of race/ethnicity and income further nuances these differences, with, for example, Black and Latinx children in the U.S. reporting higher screen time than their white peers, especially in high-income households [19].

Cultural norms also play a significant role in shaping parental mediation strategies and the types of content that children are exposed to. In collectivist societies, such as many in Asia and Latin America, family-based digital activities and stricter supervision are more common, while individualistic cultures, like those in Northern Europe and Australia, tend to emphasise autonomy and open dialogue about digital use [26,27]. Cross-cultural research demonstrates that Australian parents, for instance, are more actively engaged in all types of parental mediation compared to Singaporean parents, reflecting broader cultural values around child autonomy and psychosocial development [26]. In both contexts, higher parental digital literacy and self-efficacy are linked to more active and effective mediation strategies, but the style and intensity of mediation are shaped by cultural expectations and parental beliefs [27].

Parental mediation emerges across contexts as a key protective factor for digital wellbeing. Evidence consistently shows that active and dialogic mediation—characterised by co-use, open communication, and guidance—promotes healthier digital habits and greater resilience among children and adolescents [28,29]. The effectiveness of this mediation, however, is strongly influenced by parental digital literacy, cultural expectations, and the quality of the parent–child relationship. In collectivist societies, mediation tends to be more restrictive and protective, while in individualistic contexts, autonomy and dialogue are prioritised [26,27]. Regardless of context, empowering parents with digital competencies and fostering open communication are central strategies for mitigating digital risks and maximising opportunities.

However, it is important to recognise that active and dialogic mediation faces challenges in rapidly changing digital environments and contexts marked by significant digital divides. Continuous adaptation and support for families are, therefore, essential.

Cultural values play a decisive role in shaping how families mediate children’s digital experiences. In collectivist societies—such as those in parts of Asia and Latin America—family-based digital activities and stricter supervision are more prevalent, with parents often adopting protective or restrictive mediation strategies. This pattern is supported by cross-cultural research, which shows that parents in these contexts tend to emphasise obedience, respect for authority, and collective wellbeing, leading to more frequent use of time restrictions and content controls [26,30].

In contrast, individualistic cultures, such as those in Northern Europe and the USA, prioritise autonomy, self-regulation, and open dialogue about digital use. Here, parents are more likely to engage in co-use, encourage independent decision making, and foster open communication about online risks and opportunities [27,29].

However, the style and effectiveness of mediation are strongly influenced by cultural expectations and, crucially, by parental digital literacy. For example, in Portugal and Brazil, families with higher digital competence are more likely to adopt active mediation strategies—such as co-use and guidance—engaging in dialogue and helping children to navigate online risks. Conversely, families with lower digital literacy often rely on rigid rules or time limits, sometimes without sufficient explanation or understanding of the specific risks and opportunities associated with digital media [28,31].

This aligns with studies showing that higher parental digital literacy and self-efficacy are linked to more active and effective mediation, while lower competence is associated with more restrictive or even permissive approaches [29,32].

It is also important to note that the effectiveness of mediation is not solely determined by the type of strategy, but also by the quality of the parent–child relationship and the broader cultural context. Research in European countries, including Portugal, indicates that authoritative mediation—characterised by a balance of guidance, monitoring, and open communication—is the most effective in supporting healthy digital habits [28,33]. However, even within the same cultural context, socioeconomic status, parental education, and attitudes toward technology can lead to significant variations in mediation style and outcomes [31].

Furthermore, our findings echo the international literature in recognising that active mediation (dialogue and co-use) tends to have more beneficial effects on children’s digital experiences than purely restrictive approaches. Restrictive mediation, while protective in some respects, may limit opportunities for digital literacy and self-regulation, especially if not accompanied by open communication and guidance [28]. This is particularly relevant in the Portuguese and Brazilian contexts, where digital divides and varying levels of parental competence can influence the balance between protection and empowerment.

Political and policy contexts further influence children’s digital experiences. Countries with robust online safety frameworks and digital literacy programs tend to report higher awareness of risks and more effective parental mediation [11,13]. In contrast, in regions where such policies are less developed, children may be more exposed to online risks and less supported in developing critical digital skills. These contextual differences highlight the importance of adapting interventions and policies to local realities, rather than adopting a one-size-fits-all approach [11,13].

Our review also identified concrete examples from the included studies: in Brazil, children are more likely to access the internet via mobile devices and in private spaces, while in Portugal, there is a growing trend of daily smartphone use, but also notable digital divides among families [16,20]. During the COVID-19 pandemic, families in both Portugal and Brazil reported intensified challenges in mediating children’s digital use, with strategies ranging from restrictive (time/content limits) to active (dialogue and co-use). The effectiveness of these strategies was closely linked to parental digital literacy and cultural attitudes toward technology [20,21]. Access to the internet at school is higher in most European countries compared to Brazil, where only about one-third of children report school-based internet access, impacting both digital skills and educational opportunities [16].

These findings underscore the need for context-sensitive interventions and policies. Effective strategies must consider local realities, including socioeconomic disparities, cultural norms, and existing policy frameworks. In low- and middle-income contexts, efforts should focus on improving access, supporting parental digital literacy, and addressing digital divides [8,16]. In high-income settings, interventions may prioritise managing overuse, promoting healthy digital habits, and addressing emerging clinical concerns [5]. Across all contexts, empowering families and schools to adopt balanced, dialogic mediation strategies is essential for promoting digital wellbeing [20,21].

Concrete examples of successful interventions and best practices have been documented in the literature and can inform future efforts. At the family level, evidence supports the adoption of active and dialogic parental mediation, including co-use, open communication, and guidance. Parenting programs that train parents to discuss online risks, model healthy digital habits, and set flexible but clear rules have been shown to increase children’s digital resilience and reduce problematic behaviours [28,29]. Positive digital parenting—where parents balance supervision with support for autonomy—has been recommended as a best practice in both European and international guidelines [12,27].

In schools, structured digital literacy programs integrated into the curriculum have demonstrated improvements in academic performance, critical thinking, and safe online behaviours. Teacher training initiatives that support the pedagogical use of technology and promote active learning—such as student-led digital projects—also contribute to the development of digital and transversal skills [11,27]. The “One Laptop per Child” initiative in countries like Costa Rica has led to increased school motivation and digital competence, though its effectiveness depends on ongoing pedagogical support and family engagement [12].

For mental health and wellbeing, digital interventions such as educational apps, gamified platforms, and peer-support networks have shown moderate effectiveness in reducing anxiety and depression and in fostering socio-emotional skills [12,34]. Programs that combine technology with professional or peer guidance—like the Connected Wellbeing Initiative—enhance engagement and build resilience among youth.

At the community and policy level, national awareness campaigns and multi-sector collaborations have proven valuable. For instance, the Australian eSafety Commissioner’s initiatives and UNICEF’s digital literacy campaigns have increased awareness, improved parental, and youth skills, and promoted safer digital environments [11,12]. Policies that guarantee equitable access to devices and connectivity, alongside parental and teacher training, are crucial for reducing digital inequalities and maximising educational and social opportunities for all children.

In summary, the literature highlights that successful interventions are those that combine educational, technological, and psychosocial strategies, are adapted to the local context, and involve families, schools, professionals, and young people themselves in their design and implementation. Such integrated approaches are essential for promoting digital wellbeing and ensuring that technological advances translate into real opportunities for healthy development.

Health professionals—such as nurses, psychologists, and paediatricians—play a fundamental and complementary role in digital mediation and the promotion of digital wellbeing [34,35,36]. Nurses act as digital health educators, guiding families on safe app use, identifying early signs of problematic use, and supporting healthy practices in clinical, school, and community settings [35,36]. Psychologists contribute with evidence-based interventions for emotional regulation and socio-emotional skill development [34], while paediatricians often serve as the first point of contact for families concerned about excessive technology use, guiding healthy limits and referrals when necessary [35]. Professional mediation differs from parental mediation by involving technical knowledge, access to screening and intervention tools, and the ability to act at multiple levels (prevention, diagnosis, treatment, and advocacy) [35,36]. Interprofessional collaboration is essential for integrated and effective interventions [35]. When compared to recent systematic reviews and meta-analyses, this study confirms that well-designed digital interventions can promote wellbeing, but their effectiveness depends on context, design quality, and the involvement of parents and professionals [35,36]. This review adds to the literature by emphasising the importance of cultural and contextual adaptation, the need for multisectoral interventions, and the value of the health professional’s role in digital mediation—dimensions less explored in previous reviews [35]. While some reviews focus predominantly on app-based interventions, our findings reinforce the need to combine technological strategies with educational, psychosocial, and policy approaches, especially in more vulnerable contexts [36].

In summary, the interplay between cultural norms, parental digital literacy, and mediation style is complex and dynamic. Our results reinforce the need for context-sensitive interventions that support families in developing both the skills and the confidence to engage in active, dialogic mediation—tailored to their cultural realities and the evolving digital landscape [26,27].

### 4.2. Divergences and Paradoxes

Although most quantitative studies associate screen time with cognitive impairment, two studies [16,20] identified specific benefits, such as improved vocabulary through educational applications. This paradox calls for caution in interpretation: as highlighted by [4], the content consumed and the context of use are critical moderators that are often not controlled for in cross-sectional studies.

### 4.3. Negative Impacts of Excessive Use of Digital Technology

The results converge on a robust association between excessive screen time and damage to the physical and mental health of children and adolescents. The studies included in this review, such as [16,17,18,20,21], highlight that the prolonged use of digital devices is related to an increase in sedentary lifestyles, obesity, sleep disorders, and vision problems, mainly due to the emission of blue light and a reduction in daily physical activity [16,17,18,20,21]. Parents report concerns about the reduction in time dedicated to physical activity and sleep, factors that remain associated with physical symptoms even after adjusting for other variables [20].

In the field of mental health, the recent literature points to a significant correlation between problematic smartphone use and symptoms of anxiety, depression, and insomnia in adolescents, as demonstrated by [18,20,21]. Digital addiction and cyberbullying are emerging risks, with a direct impact on self-esteem, emotional wellbeing, and the quality of interpersonal relationships [17].

The association between excessive screen time and negative impacts on the physical, mental, and cognitive health of children and adolescents, as identified in the included studies [16,20,21], is widely corroborated by international reviews and meta-analyses. Recent studies confirm that excessive screen time is associated with a higher risk of obesity, sleep disorders, depressive symptoms, and anxiety, as well as attention difficulties and academic performance. These findings are consistent with research such as that by [5] and with data from the Adolescent Brain Cognitive Development Study, which point to moderate negative effects of screen time on multiple domains of child development.

### 4.4. Effects on Cognitive, Educational and Social Development

Inappropriate and unregulated use of digital technologies can compromise neuropsychomotor development, resulting in attention deficits, speech difficulties, language delays, and poor school performance [16,20,21]. Constant exposure to fast-paced digital content can reduce the ability to concentrate and impair academic performance, especially when screen time replaces educational activities, play, and face-to-face interactions [17,18].

However, the literature also recognises that the balanced and guided use of technologies can promote cognitive and educational benefits, especially when associated with quality educational apps and active adult mediation [16,20]. Studies show that educational apps can benefit vocabulary and literacy, but excessive and unsupervised consumption of digital content tends to harm attention, language, creativity, and academic performance [16,20,21]. Reviews such as the one by [12] and studies by [37] emphasise that video games and digital platforms can promote social skills, creativity, and emotional expression, refuting the idea that all the effects of screen time are negative.

### 4.5. Emerging Challenges and Recommendations

The discussion on the use of digital technology in childhood and adolescence cannot be dissociated from the challenges of online safety, privacy, exposure to inappropriate content, and the risks of behavioural addiction [17,18,21]. The role of educators and health professionals is fundamental in guiding families and children on safe practices, healthy boundaries, and the development of self-regulation and critical thinking skills.

The literature reinforces the need for intersectoral approaches, involving schools, families, the health system, and civil society, to ensure that technologies are allies of healthy development and not sources of vulnerability [16,20,21].

### 4.6. Limitations and Prospects

This study presents several strengths that reinforce the robustness and practical value of its findings. First, all included studies met 100% of the JBI quality criteria, ensuring a high level of methodological rigour. Second, the inclusion of research from diverse geographical and socioeconomic contexts (Brazil, USA, Indonesia, Nigeria, Croatia, and Turkey) enhances the external validity and allows for a nuanced analysis of contextual differences, an aspect often neglected in previous reviews. Third, the synthesis of concrete intervention strategies and best practices provides actionable insights for families, educators, health professionals, and policymakers, directly supporting the development of context-sensitive approaches to digital wellbeing

A critical reflection on the included studies also reveals potential sources of bias, such as reliance on self-reported data, which may be subject to recall or social desirability bias, and the underrepresentation of certain demographic groups, including children from rural areas or minority backgrounds. Furthermore, the heterogeneity of study designs, measurement instruments, and operational definitions of key variables (e.g., “screen time” and “digital wellbeing”) complicates meta-analytical synthesis and may contribute to inconsistent findings across the literature [5,8].

Despite the high methodological quality of the quantitative studies included in this review, there are several important limitations to consider. Most notably, the predominance of cross-sectional designs (6 out of 10 studies) restricts our ability to draw causal inferences. This highlights the ongoing need for longitudinal research and standardised metrics, as recommended by previous authors [38], to better understand how digital technology use impacts children and adolescents over time.

Another challenge relates to language and publication bias. By limiting our search to articles published in English and Portuguese—a decision made to expedite the review process—we may have inadvertently excluded valuable research from regions such as Asia and Africa. This, combined with the heterogeneity in assessment tools and definitions (for example, what constitutes “excessive screen time”), makes direct comparisons across studies difficult. Such variability is a well-known issue, also noted in the guidelines of the American Academy of Paediatrics [9].

To address these challenges, it is essential to invest in longitudinal and cohort studies that can track the impact of digital technology use from childhood through to adolescence. These approaches are crucial for clarifying causal relationships and identifying patterns of risk and resilience throughout development. Equally important is the development and validation of standardised instruments to assess screen time, digital engagement quality, and parental mediation strategies. Having such tools will not only facilitate comparisons across different contexts and populations, but also support more robust evidence synthesis and international meta-analyses.

A further limitation of this rapid review relates to the temporal and quantitative scope of the included studies. The decision to restrict the search to articles published between 2020 and 2025 was deliberate, aiming to capture the most up-to-date evidence in a field characterised by rapid technological evolution and shifting usage patterns among children and adolescents. While this approach ensures the relevance of the findings to current digital realities, it may have led to the exclusion of earlier studies that could provide valuable longitudinal perspectives or highlight trends that persist over time.

Additionally, the inclusion of only ten studies in the final synthesis reflects both the strict eligibility criteria—designed to ensure methodological rigour and comparability—and the inherent constraints of the rapid review methodology, which prioritises timely evidence synthesis to inform urgent decision making in health and education. While this focused approach enhances the quality and specificity of the analysis, it inevitably limits the breadth of contexts and experiences represented, potentially affecting the external validity and generalisability of the conclusions.

These limitations underscore the need for caution when interpreting the results and highlight the importance of future reviews with broader temporal windows and larger, more diverse samples. Expanding these parameters will be essential to capture the full complexity of digital technology use and its impacts across different cultural, socioeconomic, and developmental contexts.

Another priority for future research is to promote multicentre and intercultural research that includes samples from low- and middle-income countries. This approach will make it possible to capture the diversity of digital experiences, minimising publication bias and ensuring that conclusions and recommendations are relevant and applicable to different cultural and socioeconomic realities.

The integration of mixed methodologies—combining quantitative and qualitative data—is equally important. This strategy enables a deeper understanding of the family, school, and cultural contexts that influence the use and impacts of digital technologies, offering a more comprehensive and contextualised view of the phenomenon.

Finally, it is recommended that future studies systematically explore the role of protective factors such as digital literacy, active parental mediation, and the promotion of socio-emotional competences. Identifying and understanding these factors is essential for developing effective strategies that maximise the benefits of digital use and minimise the associated risks, helping to promote the wellbeing and healthy development of children and adolescents in the digital age.

### 4.7. Ethical Considerations in Digital Technology Use

The increasing integration of digital technologies in the lives of children and adolescents raises important ethical considerations. Issues such as data privacy, consent, exposure to inappropriate content, and the risk of digital addiction are particularly pressing, given that children often lack the maturity to assess online risks or understand the implications of sharing personal information [12,39]. The commercial interests driving many digital platforms can encourage prolonged engagement and may expose young users to manipulative design features, such as persuasive algorithms or in-app purchases [13]. These realities underscore the need for robust regulatory frameworks, age-appropriate digital environments, and active involvement by parents, educators, and policymakers to safeguard children’s rights and wellbeing in digital spaces [12,13].

## 5. Conclusions

This review demonstrates that digital technology use among children and adolescents is a complex phenomenon with significant effects on physical, mental, cognitive, educational, and social development. Excessive screen time is consistently linked to increased risks such as sedentary behaviour, sleep problems, anxiety, depression, and academic difficulties, although benefits can occur when digital use is actively mediated by adults for educational and social purposes.

The findings highlight the crucial role of parental mediation and socioeconomic context in digital wellbeing, underscoring the need for public policies and educational initiatives that promote digital literacy, equitable access, and the active engagement of families and schools. Methodological limitations—such as reliance on cross-sectional studies and inconsistent definitions—remain, reinforcing the need for robust longitudinal research.

A balanced, evidence-based approach, tailored to cultural and social contexts, is essential. Families should focus on active, communicative mediation and healthy digital habits. Schools must embed digital literacy and online safety into their curricula and foster strong school–family partnerships. Policymakers should ensure equitable access to technology and support integrated, evidence-based strategies. Health professionals play a key role in educating families, identifying risks early, and collaborating on interventions that support mental and digital wellbeing.

Ultimately, this study provides practical recommendations for all stakeholders and calls for continued, inclusive research to keep pace with the evolving digital landscape and ensure safe, healthy development for children and adolescents.

## Figures and Tables

**Table 1:** Structure of the research question according to the PICO model.

Element	Description	Justification
P (Problem)	Children and adolescents (2–18 years)	An age group at a critical stage of development, particularly exposed to the intensive use of digital technology.
I (Intervention)	Use of digital devices with internet access (e.g., smartphones, tablets, social networks, educational and recreational apps)	It represents the set of contemporary digital practices with a possible influence on the fields of health and education.
C (Comparison)	No use, moderate/supervised use, non-digital educational methods	It makes it possible to evaluate comparative effects and distinguish between types and intensities of use.
O (Outcome)	Effects on physical, cognitive, and emotional development; educational implications; coping strategies	It reflects the multidimensional impacts of the use of technologies on the population of children and young people.

**Table 2:** Evaluation of quantitative methodology studies according to the JBI Critical Appraisal Tools.

Study	Type of Study	JBI Tool	Score (Yes/Total)	Overall Rating	Observations
[16]	Analytical cross-sectional	JBI—Critical Appraisal Checklist for Analytical Cross-Sectional Studies	10/10 (100%)	High quality (>75% of criteria met)	
[17]	Quantitative cross-sectional	JBI—Critical Appraisal Checklist for Analytical Cross-Sectional Studies	10/10 (100%)	High quality (>75% of criteria met)	The study uses a large sample (n = 2440).
[18]	Quantitative cross-sectional	JBI—Critical Appraisal Checklist for Analytical Cross-Sectional Studies	10/10 (100%)	High quality (>75% of criteria met)	It provides rare empirical evidence in an emerging context.
[19]	Cohort	JBI—Critical Appraisal Checklist for Cohort Studies	11/11 (100%)	High quality (>75% of criteria met)	Excellent methodological rigour— large representative sample and robust analyses.

**Table 3:** Evaluation of qualitative methodology studies according to the JBI Critical Appraisal Tools.

Study	Type of Study	JBI Tool	Score (Yes/Total)	Overall Rating	Observations
[20]	Qualitative	JBI—Critical Appraisal Checklist for Qualitative research	8/11 (72.7%)	Moderate quality (50–74% of criteria met)	Some methodological limitations—small and homogeneous sample, possibility of socially desirable bias, self-reported data.
[21]	Qualitative	JBI—Critical Appraisal Checklist for Qualitative research	10/11 (90.9%)	High quality (>75% of criteria met)	Theoretical basis that gives the research analytical consistency and potential for replicability.
[22]	Qualitative	JBI—Critical Appraisal Checklist for Qualitative research	9/11 (81.8%)	High quality (>75% of criteria met)	
[23]	Qualitative and exploratory	JBI—Critical Appraisal Checklist for Qualitative research	9/11 (81.8%)	High quality (>75% of criteria met)	

**Table 4:** Evaluation of mixed-methodology studies (qualitative and quantitative) according to the JBI Critical Appraisal Tools.

Study	Type of Study	JBI Tool	Score (Yes/Total)	Overall Rating	Observations
[24]	Mixed (qualitative and quantitative)	JBI—Critical Appraisal Checklist for Mixed-Methods studies	9/9 (100%)	High quality (>75% of criteria met)	An in-depth discussion of parental mediation strategies is provided.
[25]	Mixed (qualitative and quantitative)	JBI—Critical Appraisal Checklist for Mixed-Methods studies	9/9 (100%)	High quality (>75% of criteria met)	It has a large quantitative sample (n = 582).

**Table 5:** Summary of the methodological and thematic characteristics of the included studies.

Author (Year)	Country	Type of Study	Sample (N, Age)	Context	Objectives	Thematic Category	Main Conclusions
[16]	Brazil	Quantitative, transversal, descriptive, exploratory	N = 180, 24 to 42 months	School	To analyse how early childhood screen exposure time is influenced	Child development; screen time; environmental and socioeconomic factors	Most children use digital devices, mainly television, followed by smartphones and tablets. In total, 63% have more screen time than recommended. Family factors influence this.
[20]	Croatia	Qualitative	N = 31, 4 to 8 years + 31 parents	Family, school	To describe favoured digital tech devices, activities, and emotions, and the parents’ view	Use of digital devices in childhood; parenthood; parental mediation; child wellbeing	Smartphones are children’s favourite device. They are used mainly for games, videos, and cartoons. Parents authorise digital use to promote calm and occupation, but recognise the associated risks.
[17]	Chile	Quantitative, transversal	N = 2440, 9 to 12 years	School	To describe technology use, risks, performance and satisfaction, and sleep’s role	Use of technology; school performance; sleep; life satisfaction	Using tech too much at night causes sleep issues and bad grades in teens. Lack of sleep is a big problem. Screen time does not directly affect life satisfaction. But it does if you are the victim of bullying or exposed to violent content.
[21]	Indonesia	Qualitative, exploratory	N = 22 parents of 7 to 11 children + 6 therapists	Family, clinical	To analyse parents’ and therapists’ experiences, perceptions, and opinions of digital interventions to prevent internet addiction	Digital interventions; internet addiction; parental mediation; children’s mental health	Parents and therapists recognise the potential of digital interventions to prevent and promote healthy online behaviour and strengthen parental mediation. Barriers include knowledge, time, alternatives, and privacy. Recommended strategies include personalised content, digital literacy, and targeted training.
[18]	Nigeria	Quantitative, transversal	N = 1050, 13 to 18 years + their parents	Family, school	To explore gender differences related to online risks and parental strategies	Parental mediation; digital risks; gender; teenagers; online behaviour	Girls use devices for socialisation and are more exposed to risk. Boys use different media and face different risks. Restrictive mediation is associated with lower risk, and active mediation with higher. Mothers adopt active and restrictive mediation; fathers use technical strategies influenced by their level of education. Individual vulnerabilities are key.
[19]	United States of America (USA)	Quantitative, longitudinal, national cohort	N = 9538, 9 to 10 years	School, clinical	To analyse how screen time affects mental health, using the Child Behaviour Checklist	Mental health; screen time; depression; ADHD	Screen time in adolescents is linked to depression and behavioural changes. Guidelines and early interventions can protect mental health.
[24]	USA	Mixed (qualitative and quantitative)	N = 279 parents	Family	To analyse how parents check their teenagers’ use of social media and what they think about it	Parental mediation; problematic use of technology; family relationships	Restrictive parenting correlated with problematic internet use among teenagers, while active and different strategies had no significant impact. Problematic use by parents was associated with their children’s risk. The research reveals diversity in parental approaches and a need for guidance. There is no universally effective strategy.
[22]	Indonesia	Qualitative	N = 9 male adolescents, 15 to 17 years	Family	To characterise the factors associated with Indonesian male adolescents’ internet addiction, its physical, emotional, and social impacts, and their self-control strategies	Internet dependency; social needs; self-regulation	During the COVID-19 pandemic, excessive internet use has been common as a coping strategy. Despite knowing the risks, many teens struggle to control their use, even though they recognise the problems. Strategies that limit online time and increase face-to-face contact have proved effective. Educational interventions and psychosocial support are particularly important for introverted adolescents.
[25]	Turkey	Mixed (qualitative and quantitative)	N = 582, 39 to 69 months + 20 parents	Family, school	To analyse the impact on pre-school children’s lifestyle habits of problematic technology use and eating habits, and self-care skills	Problematic use; eating behaviour; self-care; screen time	More screen time before bed can lead to problematic eating behaviours. Parents often use these devices to calm or entertain their children. However, this can harm the development of their children’s language skills, as children tend to imitate expressions from the content they see. To mitigate these effects, it is recommended that parents strengthen digital literacy and promote alternative activities and the active involvement of educators.
[23]	Croatia	Qualitative, exploratory	N = 31, 13 to 17 years	Family, school	To identify and categorise reasons for and the frequency of children’s use of digital technologies as seen by children and parents.	Use of technology; emotional wellbeing; family dynamics	Children and parents see communication and interaction as the main reasons for using technology, with entertainment a close second. Devices are used to socialise, though parents do not always recognise this. Learning is also mentioned, but less often and with less emphasis. Fear of social exclusion, boredom, and inactivity are some of the less obvious reasons. Different motives reflect universal needs in different life contexts.

**Table 6:** Summary of the main evidence from the included studies, organised by predominant thematic category.

Author (Year)	Patterns of Use of Digital Technology	Impact on Physical Health	Impact on Mental and Emotional Health	Impact on Educational Development	Impact on Social Development	Mediating and Protective Factors of Digital Wellbeing
[16]	Screen use rises from early childhood to adolescence. In total, 63% of 2–4-year-olds exceed the recommended 2 h. Television is the most popular, followed by smartphones and tablets.	Screen time is a risk factor for sedentary lifestyles, obesity, and high blood pressure from childhood onwards.		Educational apps improve vocabulary and literacy, but too much content detracts from learning.	Using technology inappropriately can harm family bonds and face-to-face interactions.	Parental supervision and family context are key to minimising risks.
[20]	Four- to eight-year-olds prefer smartphones (58.8%), followed by television, PlayStation. and computers. They play games and watch cartoons.	Parents see negative effects like sleep deprivation, sedentary lifestyles, insomnia, and visual problems.	Screen use makes people irritable, sad, and frustrated. In total, 90% of users are happy with screens, but 26% get angry or sad when they stop.	Parents value devices for education but recognise the risks.	Online communication is favoured over face-to-face interactions.	Different parental mediation strategies have different results. Online communication is preferred over face-to-face communication.
[17]	Mobile phone use rises from 4th to 7th grade, especially at weekends. In total, 28.2% play video games for over 2 h a day; 42.1% play online with strangers.	Sleep deprivation affects 12.7–23.5% of students; 72.5% of people gamble after 9 pm.	Online risk and bullying reduce satisfaction; 9.7% reported victimisation.	Overuse of phones and video games causes poor grades; sleep loss makes it worse.	Social risks include strangers, violent content. and hacking.	Sleep deprivation is key. Perceiving negative effects of night-time use protects school performance.
[21]	Children start using the internet between the ages of 1 and 5, and 82% have access to a device. Their favourite activities are watching videos and playing games.	In total, 18% of problems are eating-related and 14% are vision-related.	Unsupervised use leads to aggression, isolation, and anxiety. Risk of moderate to severe dependency.	In total, 45% of children underperform or show creativity; 32% procrastinate.	Poor communication (18%) and inappropriate language (36%) reflect social difficulties.	Supervision, relations, and literacy protect; technical and cultural issues hinder interventions.
[18]	More smartphone and social media use, especially among women for social and men for gaming.	It is linked to a sedentary lifestyle, sleep disturbances, and fatigue.	Online time and risks affect mood, self-esteem, and anxiety, especially in young women.	Excessive online use can harm school performance, especially without parental mediation.	Using social networks for too long can lead to socialisation and exclusion as well. In total, 28% feel left out because they are behind.	Parental mediation is important; the digital environment affects behaviour and risk.
[19]	The average daily screen time, excluding school use, was 4.0 ± 3.2 h. The most used devices and content were television (1.3 h/day), YouTube (1.3 h/day), and games (1.2 h/day). The use of social networks was minimal.	More screen time was linked to less physical activity and sleep. Even controlling for these factors, screen time was still linked to physical and mental health problems.	Research shows a link between more screen time and depression, behavioural disorders, ADHD, and ODD. The main digital activities linked to depression are video calls, texts, watching videos (e.g., YouTube), and gaming.	Evidence shows that ADHD, ODD, and other behavioural disorders can significantly affect children and teens’ academic performance, due to issues with focus, emotional control, and completing tasks, which interfere with their learning.	Excessive screen use is linked to bad behaviour and a lack of face-to-face interactions. This can harm social skills and interpersonal relationships in childhood and adolescence. Reduced face-to-face contact limits vital skills like empathy and emotional self-regulation.	Sleep, exercise, and ethnicity are important in the link between technology use and symptoms. Research shows this link is stronger in white children. Technology may help minority groups by offering a social and emotional support network.
[24]	Teens already use smartphones regularly and individually. Many start before 10. Most commonly used apps include Instagram, Snapchat, TikTok, online games, and forums. Motivation is peer connection and dynamics.	Parents are concerned about the impact of digital technology on children’s daily routines, especially the effect on sleep and physical health.	Loss of motivation, anxiety, and sleep disturbances have been linked to problematic Internet use in teens. Restrictive rules appear to be linked.	Overuse of the internet and related devices has a negative educational impact. Parents say this hurts attainment and performance at school. Parents limit their use to try to improve these outcomes.	Problems linked with social isolation; active parents can improve socialisation.	Protective factors include closeness, trust, autonomy, and open dialogue. Flexible and personalised strategies are better than isolated, rigid rules.
[22]	Adolescents spend over eight hours a day online. They like games, social networks, YouTube, WhatsApp, online searches, and purchases, and have a strong emotional attachment to their phones.	Reports of dizziness, headaches, blurred vision, sleep disturbances, and changes in diet.	Anxiety about not being online; anger/irritability after losing games; insecurity/low self-esteem from social comparisons.	Prolonged use affects school performance and daily routines.	Social isolation causes a preference for online contact over face-to-face, impacting family relationships.	Teenagers use strategies like deleting gaming apps, limiting mobile data, and finding alternative activities and social interaction.
[25]	Use of screens at home, school, and during travel; 31% children get ≥2 h of screen time daily, 8% get ≥4.	A sedentary lifestyle can cause headaches, sleep disorders, fever, visual problems, hyperactivity, and other health problems.	Preventing interruption; no sleep, eating and hygiene; strong emotional connection to technology; fighting against parental help.	Potential impact on education, attention, language, and cognitive performance; reduced play and manual activities, according to reports.	Mimicry, bad language, and changes in how we talk to each other.	Supervision and mediation are essential. Controlled exposure to appropriate content can benefit the child, and parental modelling influences their development. Alternative activities promote resilience and independence.
[23]	Devices are used 5–6 h a day, including phones, PlayStation, gaming, and WhatsApp. This is for communication, entertainment, research, and free time.	Associated with headaches, neck pain, and sleep disorders.	Some children experience FoMO (fear of missing out) and emotional dependence on technology.	Children use technology for schoolwork and online classes.	The main impact is social interaction, now online.	Interaction and learning are considered positive, while boredom and FoMO are negative. This can cause family conflicts, but also offer opportunities for improved communication and personalised guidance.

## Data Availability

No new data were created or analysed in this study. Data sharing does not apply to this article.

## Supplementary Materials

The following supporting information can be downloaded at: https://www.mdpi.com/article/10.3390/healthcare13141769/s1, Search Strategies.

## Abbreviations

The following abbreviations are used in this manuscript:

JBI	Joanna Briggs Institute
PICO	Population, Intervention, Comparison, Outcome
PRISMA	Preferred Reporting Items for Systematic Reviews and Meta-Analyses
WHO	World Health Organisation
