# Supplementary material for: On–Off Childhood? A Rapid Review of the Impact of Technology on Children’s Health
Source: Healthcare (Basel). 2025 Jul 21;13(14):1769. doi: 10.3390/healthcare13141769 (PMC12295127; doi:10.3390/healthcare13141769)
Supplement: Supplementary file 1 [file healthcare-13-01769-s001.zip › healthcare-3728230-supplementary.pdf]

Supplementary Material – Search Strategies

A.1. SciELO

Complete search strategy:

((children OR adolescents OR "crianças" OR "adolescentes") AND ("digital technology" OR "mobile devices" OR "smartphone" OR "internet" OR "tecnologia digital" OR "dispositivos móveis" OR "telemóvel" OR "internet")) AND ("health" OR "mental health" OR "physical health" OR "education" OR "saúde" OR "saúde mental" OR "saúde física" OR "educação") AND ("impact" OR "effects" OR "consequences" OR "implicações" OR "efeitos" OR "consequências")

Filters applied:

- Languages: Portuguese, English
- Year of publication: 2020–2025
- Document type: Scientific articles
- Full text available

A.2. B-On

Complete search strategy:

(AB(children OR adolescents OR "crianças" OR "adolescentes") AND AB("digital technology" OR "mobile devices" OR smartphone OR internet OR "tecnologia digital" OR "dispositivos móveis" OR telemóvel OR internet)) AND (AB(health OR "mental health" OR "physical health" OR education OR saúde OR "saúde mental" OR "saúde física" OR educação)) AND (AB(impact OR effects OR consequences OR implicações OR efeitos OR consequências))

Filters applied:

- Languages: Portuguese, English
- Year of publication: 2020–2025
- Document type: Peer-reviewed journals
- Full text available

Technical notes:

- The AB field indicates that the terms have been searched in the abstracts for greater precision.
